# Digital Health Interventions for Weight Management in Children and Adolescents: Systematic Review and Meta-analysis

**DOI:** 10.2196/30675

**Published:** 2022-02-14

**Authors:** Matina Kouvari, Melina Karipidou, Thomas Tsiampalis, Eirini Mamalaki, Dimitrios Poulimeneas, Eirini Bathrellou, Demosthenes Panagiotakos, Mary Yannakoulia

**Affiliations:** 1 Department of Nutrition and Dietetics School of Health Science and Education Harokopio University Athens Greece; 2 Faculty of Health University of Canberra Canberra Australia

**Keywords:** childhood obesity, eHealth, mHealth, digital health, youth, mobile phone

## Abstract

**Background:**

Recent meta-analyses suggest the use of technology-based interventions as a treatment option for obesity in adulthood. Similar meta-analytic approaches for children are scarce*.*

**Objective:**

The aim of this meta-analysis is to examine the effect of technology-based interventions on overweight and obesity treatment in children and adolescents.

**Methods:**

A systematic literature search was performed using MEDLINE (PubMed), Scopus, and Cochrane Library for randomized clinical trials to identify interventional studies published between January 2000 and February 2021.

**Results:**

In total, 9 manuscripts from 8 clinical trials of 582 children or adolescents were considered eligible. BMI, BMI z-score, and other BMI-related baseline metrics during and after intervention were considered as primary outcomes. In 7 of 8 studies, a technology-based intervention was applied in addition to conventional care. Of the 8 studies, 6 studies were conducted in the United States, 1 in Australia, and 1 in northwestern Europe. In total, 5 studies included adolescents, whereas the rest addressed children aged 9 to 12 years. Intervention duration ranged from 3 to 24 months. Significant differences between groups in BMI metric changes were reported by 5 of the 8 studies. Pooled analysis revealed an overall significant decrease in BMI metrics in the intervention group (standardized mean difference –0.61, 95% CI –1.10 to –0.13; *P*=.01). Subgroup analysis revealed that significance was lost in case of no parental involvement (standardized mean difference –0.36, 95% CI –0.83 to 0.11; *P*=.14). The small number of clinical trials found, the varying study quality, and the study heterogeneity are some limitations of this review.

**Conclusions:**

The studies reported herein describe functional and acceptable technology-based approaches, in addition to conventional treatments, to enhance weight loss in young populations.

## Introduction

### Background

Excess weight in childhood and adolescence has remained one of the most important global public health challenges since emerging as a concern several decades ago [1]. The urgent need to reverse the course of childhood obesity has led to significant growth in research regarding the efficacy of childhood obesity interventions [2]. Various interventions have been tested so far, from school-based interventions to comprehensive behavioral programs with multiple components, delivered by a multidisciplinary team [3,4]. Such models of treatment—even when effective—are often inconvenient, burdensome, and inaccessible in some cases. New computer- or mobile-assisted information and communication tools can provide useful means to develop smart digital health interventions that could tackle childhood obesity [5,6]. Data collected through internet-linked systems, electronic health records capturing clinical or demographic information, and sensors or smartphones tracking dietary behaviors provide the opportunity to generate useful knowledge regarding users’ health, behavior, and progress [7].

A previous meta-analysis with 83 randomized clinical trials (RCTs) has suggested the use of technology-based interventions as a treatment option for obesity in adulthood with potential benefits in weight loss [8]. For children and adolescents, there is only 1 meta-analysis on eHealth overweight and obesity interventions, where parents or caregivers were the agents of change [9]. The meta-analysis included interventions, such as behavioral websites with nutrition information, interactive voice response sessions, or telemedicine via videoconferencing. The fact that most of the eligible technological facilities lacked an interaction with users and the self-monitoring component is considered a limitation. Other meta-analyses examined the effect of web-based or mobile-based interventions on children commenting on modifications in obesogenic behaviors, such as sedentary lifestyle or unhealthy nutritional habits and not on core outcomes such as BMI [10,11]. Therefore, this systematic review and meta-analysis examines the effect of technology-based interventions on overweight and obesity treatment in childhood and adolescence.

### Objectives

The objective of this meta-analysis is to determine whether such interventions, delivered mostly on top of conventional care, could be more effective in improving the weight status of children or adolescents with overweight or obesity compared with conventional care or no care. The research hypothesis in this study is that technology-based interventions are effective in weight management and in case of direct comparison with conventional care, at least equivalent to conventional care.

## Methods

### Search Strategy

Following the PRISMA (Preferred Reporting Items for Systematic Reviews and Meta-Analyses) 2009 guidelines, a computer-assisted systematic literature search (not a registered protocol) was performed by 2 independent researchers (M Kouvari and M Karipidou) using MEDLINE (PubMed), Scopus, and the Cochrane Library for RCTs examining the effect of technology-based versus conventional interventions on weight management of children and adolescents with excess weight. The search strategy was mainly based on Medical Subject Headings terms as follows: *(obesity* OR *overweight* OR *body mass index* OR *weight* OR *diet* OR *nutrition)* AND *(mobile health* OR *ehealth* OR *mhealth* OR *mobile technology* OR *Internet* OR *cellular phone* OR *cellular phones* OR *smartphone* OR *telecommunications* OR *mobile applications* OR *web-based* OR *mobile apps* OR *portable electronic app* OR *portable software app* OR *text message* OR *SMS* OR *short message service* OR *portable game* OR *computers, handheld* OR *PDA* OR *personal digital assistant* OR *social media* OR *social media health* OR *Twitter* OR *tweets* OR *Facebook* OR *Instagram* OR *mobile fitness apps* OR *online social networking* OR *virtual reality* OR *avatars* OR *online gaming* OR *video games)* AND *(pediatric* OR *child* OR *adolescent* OR *youth)* AND *(clinical trial* OR *pilot study* OR *randomized controlled clinical trial)*. The search was limited to publications in English from January 1, 2000, to February 1, 2021. Reference lists of retrieved articles were also considered when these were relevant to the issue examined yet not allocated in the basic search. The relevance of the studies was assessed using a hierarchical approach based on the title, abstract, and full manuscript.

Titles and abstracts of the identified studies were independently screened by 2 researchers (M Kouvari and M Karipidou), and duplicates were removed. Full-text copies of papers were assessed for eligibility (M Kouvari and M Karipidou), with any disagreements resolved by a third researcher (EB). Data for each included study were extracted by 1 researcher (M Kouvari) and cleaned and checked by another (M Karipidou). The 2 researchers (M Kouvari and M Karipidou) extracted data using a standardized extraction form to ensure that it adequately captured trial data. For papers in which additional information was required, the corresponding authors were contacted via email.

### Selection Criteria

Studies were selected based on the inclusion and exclusion criteria presented in Textbox 1.

### Quality Assessment of Selected Studies

The quality assessment of the selected validation studies was independently implemented by 2 researchers (M Kouvari and M Karipidou) using the Consolidated Standards of Reporting Trials statement [12]. Any differences were discussed, and a decision was made by consensus.

**Inclusion and exclusion criteria.**

**Inclusion criteria**

- Study design: controlled clinical trials with at least one arm with a technology-based intervention controlled by a second arm with a conventional care intervention or without any intervention
- Sample: children and adolescents with overweight or obesity (defined through BMI or validated growth charts) aged ≤18 years
- Intervention: technology-based intervention for children or adolescents with or without parents’ or families’ support
- Outcome: BMI, BMI *z-*score, and other BMI-related metrics (eg, BMI-SD score) at baseline, during the intervention, and at the postintervention phase were considered as the primary measurements for this meta-analysis

**Exclusion criteria**

- Review articles
- Letters to editors
- Editorials
- Articles based on studies with adults
- Articles providing only feasibility or acceptance level of the applied technology-based interventions or outcomes related only to obesogenic behaviors
- Articles in which the technology-based intervention was applied only to parents
- Articles in which the control group included the use of technology
- Articles in which the technology-based intervention was not interactive with the user, for example, telemedicine or it had only an informative character, for example, a website
- Articles with inadequate statistical information

### Effect Size Measurements

The outcome of interest in this meta-analysis was the difference between the web-based intervention and the control group with regard to the potential changes from cumulative frequency distribution in BMI or BMI z-score or BMI-SD score. Studies that reported BMI-related metric results as change scores or baseline and final values; SD, SE, or CIs; and number of participants in each intervention group were included in the meta-analysis. The mean change was calculated where required, and SDs were calculated from SE or 95% CI where SD was not reported [13]. Finally, missing SDs of the changes from baseline were calculated using an imputed correlation coefficient [13].

### Data Analysis

Standardized mean difference (SMD) was used to enable the inclusion of BMI-related metrics in the same meta-analysis. In a study that reported >1 BMI metric, BMI was used. Pooled values of SMDs between the technology-based intervention and the control group and 95% CIs as the recommended summary statistics of the effect size were calculated using either a fixed or random effects model. The fixed effects model was used when sample heterogeneity was <50%, and the random effects model was used when heterogeneity was >50%. Heterogeneity assessed the null hypothesis that all studies evaluated the same effect and was evaluated using the chi-square test. Inconsistency (*I*_2_) was calculated to quantify the total variation consistent with interstudy heterogeneity, ranging from 0% to 100%. A *P* value of <.10 for the chi-square test and *I*_2_ >50% reflected a significant heterogeneity [14]. Estimates of the effect size measures were weighted by the inverse of their variances. The random effects model (DerSimonian and Laid method) was used in the presence of heterogeneity. In contrast, fixed effects models were used to calculate effect size estimates for studies that lacked heterogeneity. Subgroup analysis of prespecified groupings was performed for the following study characteristics: duration of follow-up (3-24 months), parental involvement, and type of intervention (web based vs mobile based and others). In subgroup analyses, only the last follow-up values were considered. In studies with multiple follow-ups, only the last follow-up time was considered for the estimation of the overall effect size. Possible publication bias was assessed using a contour-enhanced funnel plot of each trial’s effect size against the SE. Funnel plot asymmetry was evaluated using the Begg and Egger tests [15]. Stata software, version 14 (StataCorp LLC) was used for all statistical analyses.

## Results

### Flow of Included Studies

**Figure 1:**
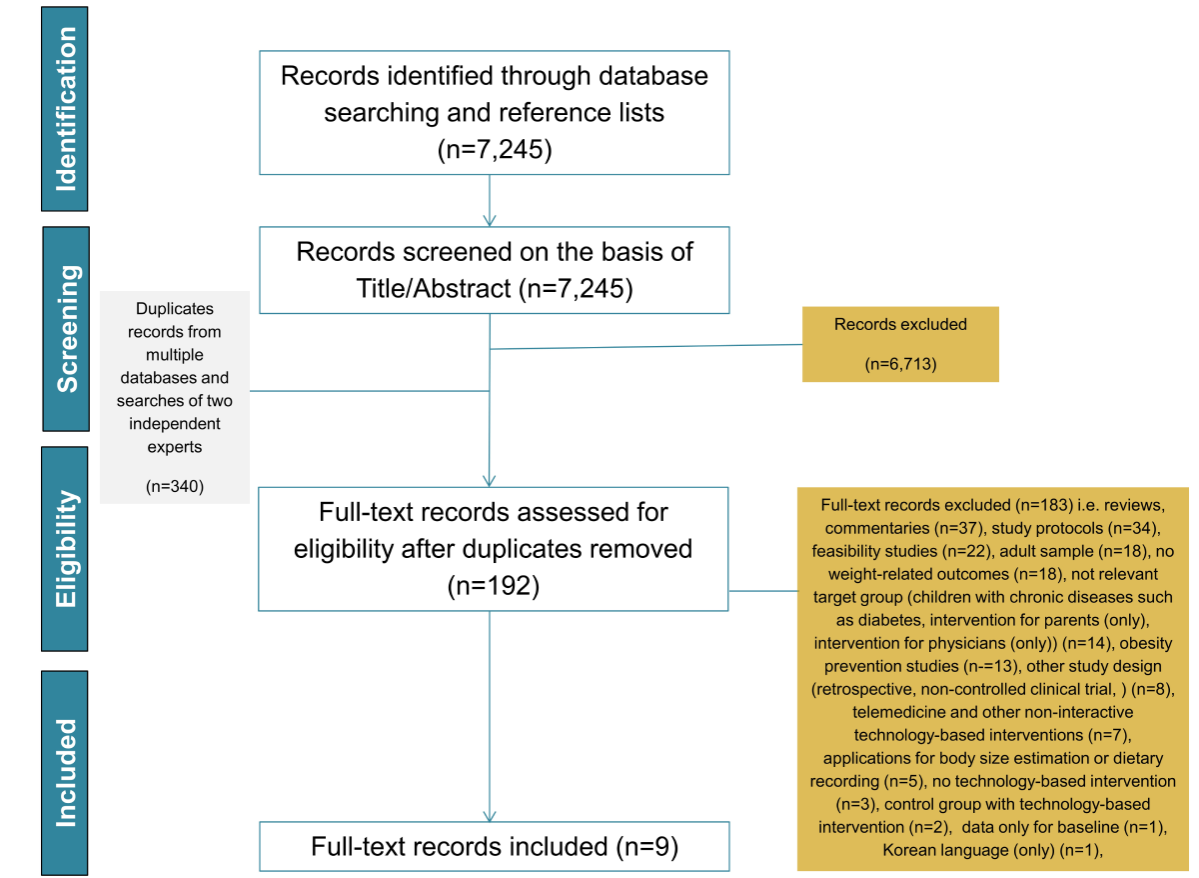
Flow diagram describing the literature review process.

A literature search flow diagram is presented in Figure 1. Initially, 7245 papers were retrieved and selected for evaluation. Then, 6713 manuscripts were removed based on their titles and abstracts as they were irrelevant to the scope of this work, accompanied by 340 duplicate records from multiple databases and searches that were also excluded. Among the rest (n=192), 9 manuscripts from 8 studies (ie, 2 separate articles were published based on 1 study regarding 2 follow-up periods) were considered relevant; 183 manuscripts were excluded, as they did not meet the inclusion criteria of this systematic review.

### General Characteristics of the Selected Clinical Trials

The characteristics of the eligible clinical trials for this meta-analysis are presented in Multimedia Appendix 1 [16-24]. In total, 582 children and adolescents participated in the selected 8 studies with a range of cultural or ethnic groups, including African American, Chinese American, White, and others. Of the 8 studies, 6 (75%) studies were conducted in the United States [16-22], 1 (13%) in Australia [23], and 1 (13%) in northwestern Europe (Netherlands) [24]. Moreover, 75% (6/8) of studies were conducted within the last decade [16-19,23,24], whereas the remaining 25% (2/8) of studies were conducted earlier [20-22]. Most of the selected studies addressed adolescents [16,17,20-23], whereas the rest had children aged 9-12 years as the target group [18,19,24]. The length of interventions ranged from 3 months [16,19,24] to 4 months [20], 6 months [17,18], and 24 months [21-23]. All studies were 2-arm controlled clinical trials, in which technology-based interventions were controlled for 1 conventional care intervention, except for 2 studies in which no intervention was implemented in the control group [16,19,24].

### Description of the Technology-Based Interventions

Of the 8 studies, 4 (50%) examined the effect of a mobile health (mHealth) intervention with or without sensors [16,17,19,24], 3 (38%) studies used a web-based intervention [18,20-22], and 1 (13%) study used an SMS text messaging intervention accompanied by telemedicine [23]. Focusing on mobile-based interventions, they also addressed nutrition-related issues and unhealthy dietary behaviors [16,17,19,24], whereas in 38% (3/8) of studies, physical activity and screen time were also taken into account [16,19,24]. In 1 study with web-based interventions, participants were enhanced to increase their physical activity level via a gamification method [18]. The other 2 web-based interventions, following a family-oriented approach, provided nutrition education accompanied by physical activity tips and counseling regarding healthy body image [20-22]. The SMS text messaging intervention along with telemedicine and group sessions focused on weight loss and weight loss maintenance, covering issues from nutrition and physical activity to body image and psychological well-being [23]. The level of parental involvement varied among the selected studies. In 6 (75%) of the 8 studies, there was participation of parents in the intervention group [18-24] accompanied by a similar participation of parents in the control group, with the exception of 2 (25%) studies [18,19]. In 7 (88%) out of 8 interventions, a hybrid approach was followed, which means that the technological tools—of any kind—were examined as supportive of conventional care treatment [17,19-24]. In 7 (88%) of the 8 studies, there was support from health care practitioners, such as dietitians, physicians, pediatricians, and psychologists [17-24]. Participants in the intervention group (children or adolescents alone or with their parents) attended weekly, biweekly, or monthly face-to-face sessions with health professionals [17,19-24] or videoconferences [18]. These sessions included goal setting, motivation techniques, individualized feedback based on the technology-based dietary or physical activity records, and enhancement to use the digital tools provided.

### Primary and Secondary Outcomes of the Selected Clinical Trials

Different measures of weight status and adiposity were used in the selected studies, with most of them using multiple measures. In total, of the 8 studies, 5 (63%) used BMI z-score [17-20,23], 3 (38%) used BMI [16,19,21,22], 3 (38%) used BMI percentiles [17,19,21,22], 2 (25%) used body fat [18,21,22], 1 (13%) used waist-to-hip ratio [23], and 1 (13%) used BMI-SD score [24]. Other metrics included modifications in obesogenic behaviors, such as dietary habits [16,18-20,22-24], physical activity habits and/or screen time [16,18,19,22,23], and physical examination or biochemical metrics [18,23]. All studies included psychological and self-efficacy metrics related to diet, physical activity, well-being, or healthy body image. With the exception of 25% (2/8) of studies [16,23], the remaining studies provided information on participants’ satisfaction and compliance with the technology-based intervention.

### Risk of Bias Within Selected Studies

The results of the risk of bias assessment for all included studies are summarized in Table 1. The selected eligible studies were of moderate quality, meeting on average, approximately 6 out of the 9 quality criteria. In particular, all studies except 1 had a well-documented randomization process [17]. In all studies, except for 1 study [22], the baseline characteristics were presented. All studies used a valid method to assess the main outcome of interest, that is, BMI, whereas only 4 (44%) out of 9 studies reported blinded assessment of the outcome of interest [16,18,20,23]. All studies except 1 [23] met the dropout rate cut-off points (ie, ≤20% for <6 months and ≤30% for ≥6 months). Regarding the quality of statistical analysis, on average, the selected studies met 2 out of 9 criteria. Specifically, all studies except 3 used intention-to-treat analysis [17,20,24]; all studies except 2 reported adequate statistical power [17,19], whereas only 4 studies provided adjusted differences between groups [16-18,23].

**Table 1:** Quality assessment of the eligible clinical trials (9 manuscripts and 8 studies)^a^.

Characteristics			Study																
			Chen et al [16]		Vidmar et al [17]		Staiano et al [18]		Wright et al [19]		Nguyen et al [23]		de Niet et al [24]		Doyle et al [20]		Williamson et al [22]		Williamson et al [21]
**Study design**																			
	Randomization described and conducted	✓				✓		✓		✓		✓		✓		✓		✓	
	Baseline characteristics by group	✓		✓		✓		✓		✓		✓		✓				✓	
**Outcome assessment**																			
	Valid measurement of BMI	✓		✓		✓		✓		✓		✓		✓		✓		✓	
	Blinded outcome assessment	✓				✓				✓				✓					
**Dropout rate**																			
	≤20% for <6 months and ≤30% for ≥6 months	✓		✓		✓		✓				✓		✓		✓		✓	
**Statistical analysis**																			
	Intention to treat for BMI outcomes	✓				✓		✓		✓						✓		✓	
	Covariates accounted for in analysis	✓				✓				✓						✓		✓	
	Power calculation reported and power adequate	✓				✓				✓		✓		✓		✓			
	Summary results, adjusted difference between groups, and CI	✓		✓		✓				✓									
**Scoring**																			
	Score in study design (range 0-2)	2		1		2		2		2		2		2		1		2	
	Score in outcome assessment (range 0-2)	2		1		2		1		2		1		2		1		1	
	Score in dropout rate (range 0-1)	1		1		1		1		0		1		1		1		1	
	Score in statistical analysis (range 0-4)	4		1		4		1		4		1		1		3		2	
	Total score (range 0-9)	9		4		9		5		8		5		6		6		6	

^a^Quality assessment was performed based on the Consolidated Standards of Reporting Trials statement.

### Separate Outcomes of Selected Studies

#### Overview

The separate outcomes of the eligible clinical trials are summarized in Multimedia Appendix 2 [16-24].

#### Weight and Adiposity Outcomes

Of the 8, 5 (63%) studies reported significant differences between groups in BMI metrics from baseline to the end of intervention [18,20,21,23,24]. The intervention duration of these studies was >6 months, and all of them addressed not only children or adolescents but also their parents. Significant reductions in body fat [22] and waist-to-hip ratio [23] were observed in interventions with a 2-year duration.

#### Diet-Related Outcomes

The 7 studies reporting modifications on dietary intake and behaviors revealed a significant difference between groups with regard to improvement in at least one dietary outcome. In particular, a decrease in consumption of sugar-sweetened beverages [16], lower carbohydrate intake [18], increased fruit consumption [19], decreased meat and fruit juice intake [23], better adherence to a healthier dietary pattern [20,24], and lower consumption of food products with high-fat content [21,22] were observed.

#### Physical Activity–Related Outcomes

Among the 5 studies that provided input on changes in participants’ physical activity level, 1 (20%) study revealed a significant decrease in screen time [16], whereas the remaining 4 (80%) studies highlighted increases in physical activity level in terms of hours per day or the intensity of exercise [17,20,21,24].

#### Physical Examination and Biochemical Metrics

In 1 (50%) of the 2 studies with physical examination and biochemical measurements, significant reductions in blood pressure and cholesterol levels were observed [18].

#### Psychological Health–Related Outcomes

All studies provided input on the effect of technology-based intervention over the control group on participants’ psychological health. Of the 8 studies, 7 (88%) studies observed that participants in the intervention group increased their self-efficacy in relation to diet [16,19,20] and physical activity [16,18,20], decreased unhealthy eating behaviors related to dieting or weight or body image [22,23], and ameliorated their self-esteem [23,24].

#### Usability and Acceptability of the Technology-Based Intervention

Of the 6 studies providing information on the level of compliance of participants with the technology-based intervention, 5 (83%) reported moderate to high levels of usability and acceptability of the technology-based intervention [17-20,24]. Nevertheless, the added value of the technology-based intervention over typical care was not clear considering the similar dropout rates between the 2 groups (dropout rate in the intervention group: 12.9%, range 0%-41%, vs dropout rate in the control group: 12.6%, range 0%-36%; *P*=.96), excluding studies in which the control group had no intervention [18,19]. Among the 6 studies, 1 (17%) revealed that children assigned to the technology-based intervention receiving SMS text messages were less likely to withdraw from the study than children who did not receive this service [24].

### Synthesis of BMI-Related Outcomes

#### Overview

**Figure 2:**
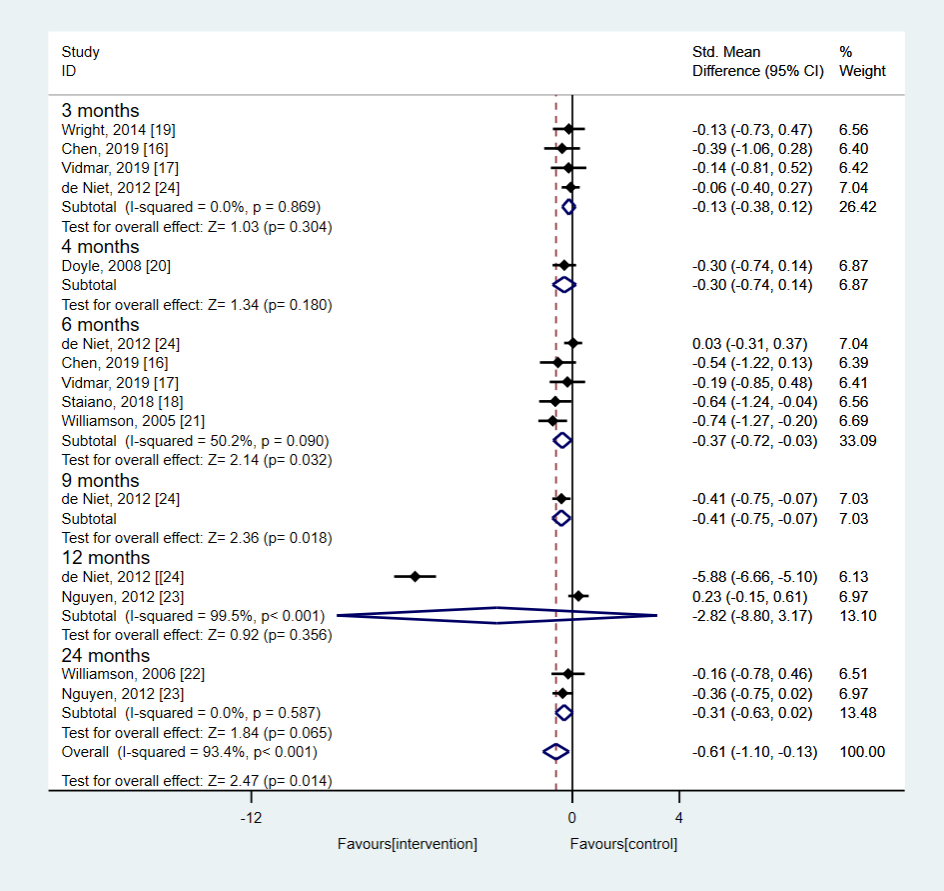
Results from the random effects meta-analysis concerning the effect of the technology-based interventions on BMI-related metrics according to the study follow-ups. In case of studies with multiple follow-ups, only the last follow-up time was considered for the estimation of the overall effect size.

**Figure 3:**
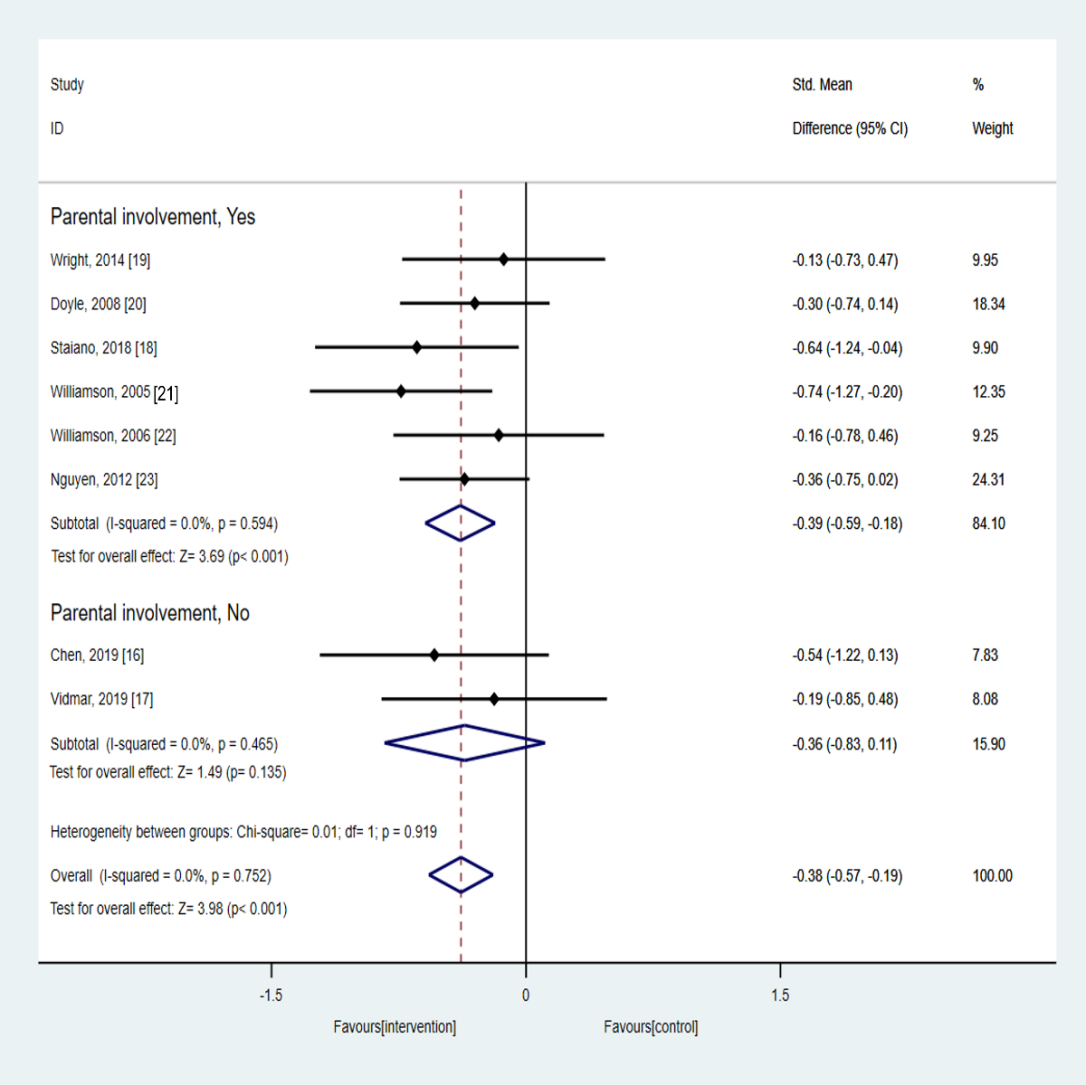
Results from the subgroup analysis according to the parental involvement of the technology-based intervention concerning its effect on BMI-related metrics. In case of studies with multiple follow-ups, only the last follow-up time was considered for the estimation of the overall effect size.

**Figure 4:**
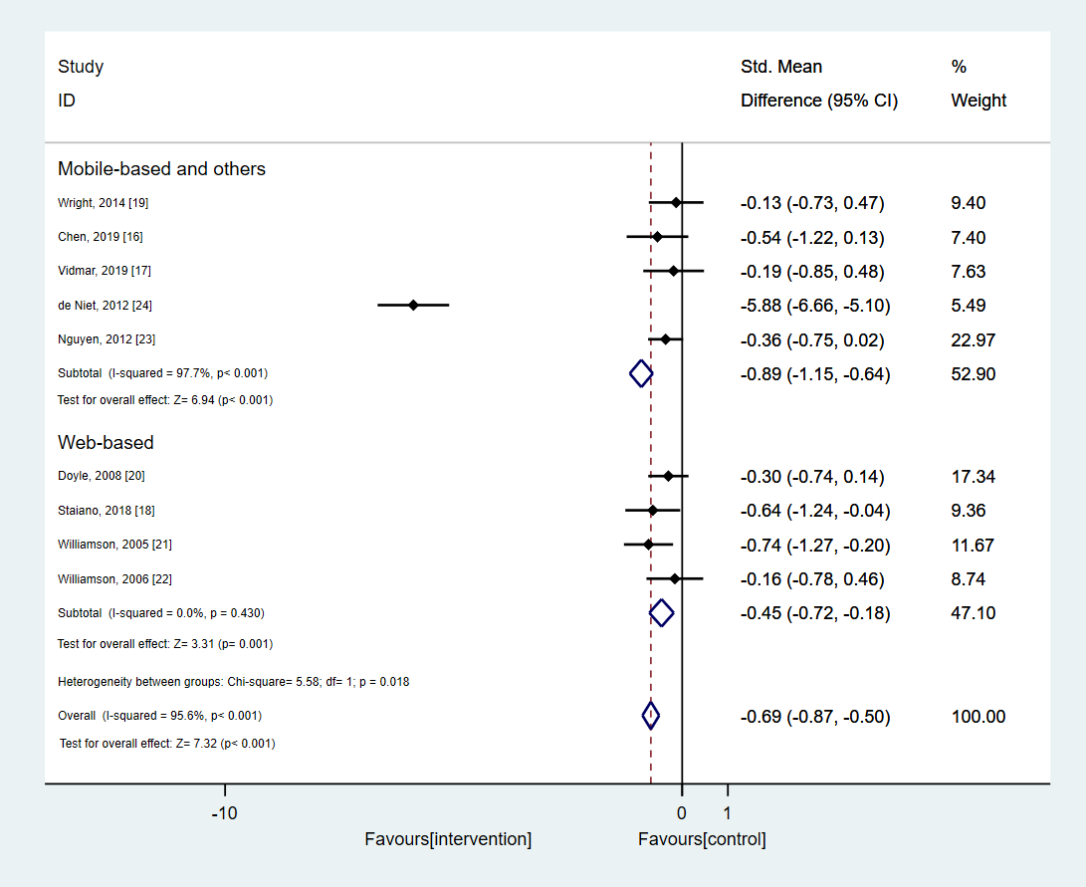
Results from the subgroup analysis according to the type of the intervention concerning its effect on BMI-related metrics. In case of studies with multiple follow-ups, only the last follow-up time was considered for the estimation of the overall effect size.

A meta-analysis was conducted on pooled data from 9 manuscripts (8 studies in total), which compared technology-based intervention groups with control groups. The meta-analysis results are presented in Figures 2-4.

As presented in Figure 2, a significantly higher decrease in the BMI-related metric was observed (SMD –0.61, 95% CI –1.10 to –0.13; *P*=.01). Compared with the other follow-ups, this was more evident after a 6-month follow-up in the technology-based intervention group when compared with the control group (SMD –0.37, 95% CI –0.72 to –0.03; *P*=.03), whereas a favorable effect of the technology-based interventions was also found after 24 months; however, statistical significance was not reached (SMD –0.31, 95% CI –0.63 to 0.02; *P*=.07).

#### Sensitivity Analysis

Of the 8 studies, 2 (25%) had a control group without any intervention. Hence, we repeated the aforementioned analysis, excluding these 2 studies. The overall outcome remained significant (SMD –0.65, 95% CI –1.20 to –0.10; *P*=.02), whereas the 6-month outcome remained marginally significant (SMD –0.32, 95% CI –0.71 to 0.07; *P*=.10).

A subgroup analysis was conducted based on parental involvement, and the results are shown in Figure 3. The meta-analysis revealed a significantly higher decrease in the BMI-related metric in the technology-based intervention group than in the control group only in case of parental involvement (SMD –0.39, 95% CI –0.59 to –0.18; *P*<.001). In the case of no parental involvement, no significant difference between groups was observed (SMD –0.36, 95% CI –0.83 to 0.11; *P*=.14).

Another subgroup analysis performed in this study was related to the type of technology-based intervention used. The results are shown in Figure 4. Interventions were grouped as web-based, mobile-based, and others. A statistically significant decrease in the BMI-related metric in the intervention group compared with that in the control group was observed both in the case of mobile-based and other interventions (SMD –0.89, 95% CI –1.15 to –0.64; *P*<.001) as well as in the case of the web-based interventions (SMD –0.45, 95% CI –0.72 to –0.18; *P*=.001).

## Discussion

### Principal Findings

This meta-analysis revealed that an intervention that combines conventional care with technological facilities could be an effective method for weight management of children and adolescents with overweight or obesity and is probably more effective than conventional care alone. These observations were more evident in the case of interventions lasting at least six months. The selected studies included eHealth and mHealth technologies, such as interactive web platforms, mobile apps, gaming, and SMS text messaging with or without sensors and were accompanied or not accompanied by other contact forms such as telemedicine, emails, and informative websites. The focus of these technologies was more or less related either exclusively or in combination with improvement in dietary habits, enhancement of physical activity, or the increasing of users’ self-monitoring potential. The type of technological means used in each intervention, that is, mobile-based or web-based, did not seem to alter the final outcome. Parental involvement was related to greater outcomes of the intervention, particularly in children; however, it was not possible to isolate the separate contribution of parents to the final outcome.

### Strengths and Limitations

To the best of our knowledge, this is the first meta-analysis to examine the effect of technology-based interventions on weight management in childhood and adolescence using strict eligibility criteria, such as the existence of a control—without any kind of technology—group, the exclusion of technology-based interventions without an interactive—with the user—character, and the exclusion of studies providing the effect on weight-related metrics of normal-weight children or adolescents (obesity prevention spectrum). However, several limitations exist, including the restriction to articles published only in English, the small number of clinical trials found with varying study quality, heterogeneity of the studies, inadequacy of the power to detect an outcome in some studies because of the small number of participants, varying aims between studies, and all but 2 studies being conducted in the United States. Finally, no definite conclusion can be drawn on whether the actual difference in weight management was caused by the different techniques used or by the differences in other intervention characteristics (eg, number of sessions).

### Comparison With Prior Work

To date, technology-based health interventions for weight management in young people have been reported as a rapidly developing research area; although promising results have been produced, more research is definitely in order [25]. Hitherto, studies have described technologies with the potential to promote healthy behaviors related to nutrition or physical activity in children and adolescents [26,27]. The pooled higher reduction in adiposity metrics observed in this study in favor of technology-based intervention groups were observed with improvements in users’ dietary habits, physical activity level, and screen time and with better psychological health regarding body image, self-esteem, or weight- and dieting-related stress. Examining the actual efficiency and added value of technology-based methods over conventional interventions in childhood obesity management remains a challenging research field for many reasons. Most interventional studies combine eHealth or mHealth interventions with other mostly conventional treatments (hybrid approach), thus making the efficacy of technology-only approaches to affect adiposity outcomes difficult to ascertain [9,26]. In most studies selected for this meta-analysis, the digital behavior change intervention was examined on top of conventional care and matched for conventional-only care, revealing an advantage of the former in weight loss. On the other hand, digital behavior change interventions have not been generated to replace the role of health professionals and the multifaceted treatments required for management of excess weight in childhood and adolescence but rather to support them, showing potential as an additional tool for patient monitoring and designing tailor-made interventions through the selection of more valid information and plausibly weight loss maintenance in the postintervention phase [28,29].

Another issue examined in this study was related to parental involvement in technology-based interventions. Active enrollment of parents was reported in 6 (75%) out of 8 studies [18-24], accompanied by a similar participation in the control group, with the exception of 2 studies [18,19]. Interestingly, 2-8 studies without parental involvement did not achieve significant modifications in adiposity metrics. Although many reasons could be responsible for this nonsignificance, such as the fact that lower combined sample size in the 2 studies could lead to greater CIs and higher *P* values, this finding may imply that the participation of families in childhood obesity management programs remains of high importance even in or especially in interventions with advanced technological means. Currently, interventions that target parents to tackle obesity in early life stages are presented as effective, especially when it comes to preschoolers, that is, children <5 years [30-33]. On the other hand, the studies in this meta-analysis were designed for children >9 years and principally adolescents, which may challenge the level of parental involvement. Preschool-aged children are rarely targeted in such technology-based interventions. The MINISTOP RCT is probably the very first study to apply a 6-month technology-based weight loss intervention to children <5 years, using their parents as the primary target group. Although no significant differences in adiposity metrics between the control and intervention groups were observed, children and parents assigned to the technology-based group seemed to significantly ameliorate their nutrition and physical activity habits [34]. Considering the fact that technological approaches and parental involvement are usually presented as effective practices to tackle excess weight in childhood, their combination into 1 tailor-made weight loss intervention may result in multiple positive outcomes.

The studies in this meta-analysis provided input on the usability and acceptability of technology-based interventions. Most of them reported a moderate to high level of adherence to the intervention using different criteria and metrics, such as the level of user enjoyment, the frequency of app use, or the number of SMS text messages received [17-20,24]. Nevertheless, based on the dropout rates, no significant differences were observed between the intervention and control groups in most cases. This evidence regarding the usability of technology-based interventions is based largely on the use of SMS text messaging using a mobile- or web-based approach. However, the latest technological advances include the emergence of smartphone apps [29], interactive platforms [35], and exergaming [36]. Such facilities have increased in popularity, offering a unique opportunity to implement large-scale obesity treatment interventions in youth [37]. The current orientation for improving adherence to treatments in pediatrics focuses on motivation, problem-solving skills, and reduction of posttreatment influence, resorting to several novel youth-friendly technological approaches [26]. For instance, studies have described the best placement and accuracy of mobile devices and sensors to record dietary intake or physical activity and ways to lessen user burden [26]. Reward-type incentives, provision of social connections and multiplayer capabilities, short- and long-term motivational techniques, and personalized feedback are also suggested as means to enhance user acceptability, efficiency of the intervention, and probably maintenance of positive outcomes even in the postintervention phase [26]. Focusing on gamification, many video games have been created with the aim to modify children’s or adolescents’ dietary habits or physical activity status, such as *Let’s Move! (To move!)*, *Counting Carbohydrates with Lenny*, *LeapBand*, or *Zamzee,* where users interact with virtual characters that—creating a fascinating environment—enhance them to complete a series of relevant activities and challenges [38]. Finally, early involvement of key stakeholders in the intervention development stage seems to be detrimental for the delivery of a technological tool that will be well-accepted by the target group—even more when it comes to younger ages [39].

### Conclusions

Studies reported herein describe functional and acceptable technology-based approaches, on top of conventional care, to enhance weight loss in overweight or obese children and adolescents through the promotion of a healthy lifestyle and improvement of users’ well-being. However, the large heterogeneity in study designs, settings, intervention components, and outcomes probably eliminates the strength of this conclusion. Finally, this field is advancing so quickly that the technology used is often no longer state of the art; interventions that use the full range of novel technologies, such as ubiquitous sensing and real-time feedback, are currently being developed and pilot tested. Therefore, similar meta-analytic approaches should be repeated on a regular basis.

## Appendix

Multimedia Appendix 1 Characteristics of the eligible clinical trials of the meta-analysis (9 manuscripts and 8 studies).

Multimedia Appendix 2 Primary and secondary outcomes of the eligible clinical trials of the meta-analysis (9 manuscripts and 8 studies).

## Abbreviations

mHealth: mobile health
PRISMA: Preferred Reporting Items for Systematic Reviews and Meta-Analyses
RCT: randomized clinical trial
SMD: standardized mean difference

## References

[1] Lobstein T, Jackson-Leach R, Moodie ML, Hall KD, Gortmaker SL, Swinburn BA, James WP, Wang Y, McPherson K (2015). Child and adolescent obesity: part of a bigger picture. Lancet.

[2] Di Cesare M, Sorić M, Bovet P, Miranda JJ, Bhutta Z, Stevens GA, Laxmaiah A, Kengne A, Bentham J (2019). The epidemiological burden of obesity in childhood: a worldwide epidemic requiring urgent action. BMC Med.

[3] Scott-Sheldon LA, Hedges LV, Cyr C, Young-Hyman D, Khan LK, Magnus M, King H, Arteaga S, Cawley J, Economos CD, Haire-Joshu D, Hunter CM, Lee BY, Kumanyika SK, Ritchie LD, Robinson TN, Schwartz MB (2020). Childhood obesity evidence base project: a systematic review and meta-analysis of a new taxonomy of intervention components to improve weight status in children 2-5 years of age, 2005-2019. Child Obes.

[4] Wang Y, Cai L, Wu Y, Wilson RF, Weston C, Fawole O, Bleich SN, Cheskin LJ, Showell NN, Lau BD, Chiu DT, Zhang A, Segal J (2015). What childhood obesity prevention programmes work? A systematic review and meta-analysis. Obes Rev.

[5] Lau PW, Lau EY, Wong DP, Ransdell L (2011). A systematic review of information and communication technology-based interventions for promoting physical activity behavior change in children and adolescents. J Med Internet Res.

[6] Tate EB, Spruijt-Metz D, O'Reilly G, Jordan-Marsh M, Gotsis M, Pentz MA, Dunton GF (2013). mHealth approaches to child obesity prevention: successes, unique challenges, and next directions. Transl Behav Med.

[7] Smith AJ, Skow A, Bodurtha J, Kinra S (2013). Health information technology in screening and treatment of child obesity: a systematic review. Pediatrics.

[8] Hutchesson MJ, Rollo ME, Krukowski R, Ells L, Harvey J, Morgan PJ, Callister R, Plotnikoff R, Collins CE (2015). eHealth interventions for the prevention and treatment of overweight and obesity in adults: a systematic review with meta-analysis. Obes Rev.

[9] Hammersley ML, Jones RA, Okely AD (2016). Parent-focused childhood and adolescent overweight and obesity eHealth interventions: a systematic review and meta-analysis. J Med Internet Res.

[10] Shin Y, Kim SK, Lee M (2019). Mobile phone interventions to improve adolescents' physical health: a systematic review and meta-analysis. Public Health Nurs.

[11] Champion KE, Parmenter B, McGowan C, Spring B, Wafford QE, Gardner LA, Thornton L, McBride N, Barrett EL, Teesson M, Newton NC, Health4Life team (2019). Effectiveness of school-based eHealth interventions to prevent multiple lifestyle risk behaviours among adolescents: a systematic review and meta-analysis. Lancet Digit Health.

[12] Schulz KF, Altman DG, Moher D, CONSORT Group (2010). CONSORT 2010 statement: updated guidelines for reporting parallel group randomized trials. Ann Intern Med.

[13] Cumpston M, Li T, Page M, Chandler J, Welch V, Higgins J, Thomas J (2019). Updated guidance for trusted systematic reviews: a new edition of the Cochrane Handbook for Systematic Reviews of Interventions. Cochrane Database Syst Rev.

[14] Lau J, Ioannidis JP, Schmid CH (1997). Quantitative synthesis in systematic reviews. Ann Intern Med.

[15] Irwig L, Macaskill P, Berry G, Glasziou P (1998). Bias in meta-analysis detected by a simple, graphical test. Graphical test is itself biased. Br Med J.

[16] Chen J, Guedes CM, Lung AE (2019). Smartphone-based healthy weight management intervention for Chinese American adolescents: short-term efficacy and factors associated with decreased weight. J Adolesc Health.

[17] Vidmar AP, Pretlow R, Borzutzky C, Wee CP, Fox DS, Fink C, Mittelman SD (2019). An addiction model-based mobile health weight loss intervention in adolescents with obesity. Pediatr Obes.

[18] Staiano AE, Beyl RA, Guan W, Hendrick CA, Hsia DS, Newton RL (2018). Home-based exergaming among children with overweight and obesity: a randomized clinical trial. Pediatr Obes.

[19] Wright JA, Phillips BD, Watson BL, Newby PK, Norman GJ, Adams WG (2013). Randomized trial of a family-based, automated, conversational obesity treatment program for underserved populations. Obesity (Silver Spring).

[20] Doyle AC, Goldschmidt A, Huang C, Winzelberg AJ, Taylor CB, Wilfley DE (2008). Reduction of overweight and eating disorder symptoms via the internet in adolescents: a randomized controlled trial. J Adolesc Health.

[21] Williamson DA, Martin PD, White MA, Newton R, Walden H, York-Crowe E, Alfonso A, Gordon S, Ryan D (2005). Efficacy of an internet-based behavioral weight loss program for overweight adolescent African-American girls. Eat Weight Disord.

[22] Williamson DA, Walden HM, White MA, York-Crowe E, Newton RL, Alfonso A, Gordon S, Ryan D (2006). Two-year internet-based randomized controlled trial for weight loss in African-American girls. Obesity (Silver Spring).

[23] Nguyen B, Shrewsbury VA, O'Connor J, Steinbeck KS, Hill AJ, Shah S, Kohn MR, Torvaldsen S, Baur LA (2013). Two-year outcomes of an adjunctive telephone coaching and electronic contact intervention for adolescent weight-loss maintenance: the Loozit randomized controlled trial. Int J Obes (Lond).

[24] de NJ, Timman R, Bauer S, van den Akker E, Buijks H, de KC, Kordy H, Passchier J (2012). The effect of a short message service maintenance treatment on body mass index and psychological well-being in overweight and obese children: a randomized controlled trial. Pediatr Obes.

[25] Tully L, Burls A, Sorensen J, El-Moslemany R, O'Malley G (2020). Mobile health for pediatric weight management: systematic scoping review. JMIR Mhealth Uhealth.

[26] Turner T, Spruijt-Metz D, Wen CK, Hingle MD (2015). Prevention and treatment of pediatric obesity using mobile and wireless technologies: a systematic review. Pediatr Obes.

[27] Lee J, Piao M, Byun A, Kim J (2016). A systematic review and meta-analysis of intervention for pediatric obesity using mobile technology. Stud Health Technol Inform.

[28] Partridge SR, Redfern J (2018). Strategies to engage adolescents in digital health interventions for obesity prevention and management. Healthcare (Basel).

[29] Chaplais E, Naughton G, Thivel D, Courteix D, Greene D (2015). Smartphone interventions for weight treatment and behavioral change in pediatric obesity: a systematic review. Telemed J E Health.

[30] Golley R, Hendrie G, Slater A, Corsini N (2011). Interventions that involve parents to improve children's weight-related nutrition intake and activity patterns - what nutrition and activity targets and behaviour change techniques are associated with intervention effectiveness?. Obes Rev.

[31] Yavuz HM, van Ijzendoorn MH, Mesman J, van der Veek S (2015). Interventions aimed at reducing obesity in early childhood: a meta-analysis of programs that involve parents. J Child Psychol Psychiatry.

[32] Young KM, Northern JJ, Lister KM, Drummond JA, O'Brien WH (2007). A meta-analysis of family-behavioral weight-loss treatments for children. Clin Psychol Rev.

[33] Niemeier BS, Hektner JM, Enger KB (2012). Parent participation in weight-related health interventions for children and adolescents: a systematic review and meta-analysis. Prev Med.

[34] Nyström CD, Sandin S, Henriksson P, Henriksson H, Trolle-Lagerros Y, Larsson C, Maddison R, Ortega FB, Pomeroy J, Ruiz JR, Silfvernagel K, Timpka T, Löf M (2017). Mobile-based intervention intended to stop obesity in preschool-aged children: the MINISTOP randomized controlled trial. Am J Clin Nutr.

[35] Leung MM, Mateo KF, Verdaguer S, Wyka K (2018). Testing a web-based interactive comic tool to decrease obesity risk among minority preadolescents: protocol for a pilot randomized control trial. JMIR Res Protoc.

[36] Dias JD, Domingues AN, Tibes CM, Zem-Mascarenhas SH, Fonseca LM (2018). Serious games as an educational strategy to control childhood obesity: a systematic literature review. Rev Lat Am Enfermagem.

[37] Nguyen B, Kornman KP, Baur LA (2011). A review of electronic interventions for prevention and treatment of overweight and obesity in young people. Obes Rev.

[38] Del Río NG, González-González CS, Martín-González R, Navarro-Adelantado V, Toledo-Delgado P, García-Peñalvo F (2019). Effects of a gamified educational program in the nutrition of children with obesity. J Med Syst.

[39] van der Aa DA, Altenburg TM, van Randeraad-van der Zee CH, Chinapaw MJ (2017). The effectiveness and promising strategies of obesity prevention and treatment programmes among adolescents from disadvantaged backgrounds: a systematic review. Obes Rev.

