# Supplementary material for: Digital Health Interventions for Weight Management in Children and Adolescents: Systematic Review and Meta-analysis
Source: J Med Internet Res. 2022 Feb 14;24(2):e30675. doi: 10.2196/30675 (PMC8887634; doi:10.2196/30675)
Supplement: Multimedia Appendix 1 [file jmir_v24i2e30675_app1.docx]

| **Table 1**  Characteristics of the eligible clinical trials of the meta-analysis (n=9; 8 studies ). | | | | | | | | | | | | | |
| --- | --- | --- | --- | --- | --- | --- | --- | --- | --- | --- | --- | --- | --- |
| **Study** | **Setting** | **Total (intervention/control)** | **Boys/girls, n** | **Age (years), mean (SD)** | **Intervention** | **Type of technology used** | **Hybrid interventiona** | **Control** | **Duration of intervention** | **Follow-up** | **Primary outcomes** | **Secondary outcomes** | **Information on satisfaction for the technology-based intervention** |
| **Chen et al [16]** | USA | 40 (23/17) | 23/17 | 14.9 (1.67) | **Type:** wearable sensor (Fitbit Flex) accompanied by app plus online educational modules plus text messaging; **Scope:** self-efficacy in healthy food choices and increasing physical activity; **Short description:** Track physical activity, sedentary activity, and dietary intake progress, set realistic individualized goals and monitor progress, provide tips for everyday activities and provide strategies for maintaining healthy weight;  **Behavioral change theory:** social cognitive theory  **Contribution of parents/family:** no  **Contribution of health care practitioners:** no (only for anthropometric measurements) | mobile-based; sensors; short message service | no | **Content:** Omron HJ-105 pedometer and a blank food-and-activity diary to self-monitor their dietary habits and physical activity level; **Contribution of parents/family:** no | 3 months | 3 months; 6 months | ΒΜΙ | waist-to-hip ratio; physical activity; screen time; dietary habits (fruits, vegetables, soda and sweetened beverages, breakfast, out-of-home eating); pediatric quality of life; Physical activity self-efficacy and healthy eating self-efficacy | no |
| **Vidmar et al [17]** | USA | 35 (18/17) | 14/21 | 14.3 (1.77) | **Type:** interactive, addiction‐based, weight loss smartphone app with coaching; **Scope:** staged withdrawal from participant identified problem foods, staged withdrawal from snacking between meals, withdrawal from excessive amounts of food consumed at meals **Short description:** the intervention was implemented through an iPhone® app called W8Loss2Go©, securely integrated with a network‐server for real‐time data access and storage. The intervention included two clinic visits at 3 and 6‐month intervals, short message service and weekly phone sessions.  **Contribution of parents/family:** no  **Contribution of health care practitioners:** yes (monthly clinic visits with goal setting) | mobile-based; short message service | yes | **Content:** personal sessions; **Contribution of parents/family:** no | 6 months | 3 months; 6 months | BMI z-score; %BMI_P95_ | adherence with the app intervention components; compliance with scheduled weekly phone calls; compliance with in-clinic visits; (Yale Food Addiction Scale-children) YFAS-c score | yes |
| **Staiano et al [18]** | USA | 46 (23/23) | 26/20 | 11.2  (0.8) | **Type:** GameSquad plus smartwatch Fitbit Zip ; **Scope:** increase physical activity, 60 minutes/day of moderate/vigorous physical activity; **Short description:** exergames three days/week;  **Behavioral change theory:** social cognitive theory  **Contribution of parents/family:** yes  **Contribution of health care practitioners:** yes (fitness coach over videochat in weekly or biweekly basis) | web-based; gamification; Sensors | yes | **Content:** No intervention; participants were asked to keep their typical physical activity level; **Contribution of parents/family:** no | 24 weeks | 24 weeks | ΒΜΙ z-score | SBP; DBP; LDL-C; weight z-score; fat mass; % fat mass; BMD; glucose; HDL-C; dietary intake (calorie intake, macronutrients intake) | yes |
| **Wright et al [19]** | USA | 50 | n/a | 9-12 | **Type:** telephone counseling intervention delivered by an automated interactive voice response system. The intervention also included an electronic health record behavioral counseling tool; **Scope:** monitor, educate and counsel parents and children on healthy weight management and television time; **Short description:** *Child intervention:* media program to learn the Traffic Light diet, learn about rules, learn how to self-monitor weight, food, and screen-time and set up contracts and rewards - *Parent intervention:* media program to create a home that supports health, be a good role model, build a respectful relationship with the child, and use praise and encouragement to motivate the child to do healthy things;  **Behavioral change theory:** social cognitive theory  **Contribution of parents/family:** yes  **Contribution of health care practitioners:** yes (electronic health record behavioral counseling tool used by the primary care clinician during well-child follow-up visits) | mobile-based | yes | **Content:** no intervention/wait-list control condition; **Contribution of parents/family:** no | 12 weeks | 12 weeks | initial efficacy, acceptability and feasibility of the intervention | BMI; BMI z-score; BMI percentile; parent BMI; dietary habits; parent dietary habits; TV time | yes (primary outcome) |
| **Nguyen et al [23]** | Australia | 151 (73/78) | Not available | 13-16 | **Type:** adolescent booster group sessions plus additional therapeutic contact via short message service; **Scope:** weight loss maintenance; **Short description:** seven weekly group sessions plus 4 telephone coaching sessions and 32 SMS and email messages;  **Behavioral change theory:** social cognitive theory  **Contribution of parents/family:** yes  **Contribution of health care practitioners:** yes (weekly group sessions with trained dietitians) | short message service and telemedicine | yes | **Content:** adolescent booster group sessions; **Contribution of parents/family:** yes | 24 months | 12 months; 24 months | BMI z-score | psychological well being; body image; eating behaviors; physical activity; sedentary behavior | no |
| **de Niet et al [24]** | Netherlands | 141 (73/68) | 51/90 | 9.9  (1.3) | **Type:** Big Friends' Club (BFC) program, multidisciplinary behavioural group intervention for children, plus short message service intervention; **Scope:** reduction of BMI-SDS through (i) promoting social support; (ii) motivating participants; (iii) reinforcing positive changes; and (iv)suggesting behaviour modification and self-management skills that were learnt during the BFC programme; **Short description:** Selecting children's weekly self-monitoring data on exercise and eating behaviour and mood via mobile phones. In return, children received tailored feedback messages;  **Behavioral change technique/strategy:** self-monitoring  **Contribution of parents/family:** yes  **Contribution of health care practitioners:** yes (behavioral lifestyle intervention with a group of health care professionals | mobile-based; short message service | yes | **Content:** BFC program without short message service; **Contribution of parents/family:** yes | 12 months | 3 months;, 6 months; 9 months; 12 months | BMI-SDS; eating behavior; psychological well-being | adherence to technology-based intervention | yes (secondary outcome) |
| **Doyle et al [20]** | USA | 80 (40/40) | 30/50 | 14.5  (1.7) | **Type:** Student Bodies (SB2), Internet-delivered program using a cognitive-behavioral approach; **Scope:** weight loss and positive body image; **Short description:** SB2 weekly content includes basic education (e.g., portion sizes, recommended daily activity), guided behavior modification for weight control (e.g., self-monitoring with feedback), and cognitive exercises for improving body image. A monthly newsletter was mailed to parents to encourage creating a positive and constructive home environment;  **Contribution of parents/family:** yes  **Contribution of health care practitioners:** yes (clinical psychology doctoral student sent individualized feedback counseling messages) | web-based; short message service | yes | **Content:** color handouts containing basic information on nutrition and physical activity to children and their families; **Contribution of parents/family:** yes | 16 weeks | 16 weeks (post-intervention); 4 months after the end of intervention | BMI z-score; Eating Disorder Examination-Questionnaire | frequency of adolescent behavioral and cognitive skills use related with eating and physical activity habits | yes |
| **Williamson et al [22]** | USA | 57 (29/28) | 0/57 | 13.2  (1.4) | **Type:** web-based weight management programs plus face-to-face counselling; **Scope:** weight loss via nutrition education; **Short description:** the web site provided nutrition education, interactive components such as weight graphs and behavior modification for adults and adolescents using a family‐oriented format. Counseling for behavior modification was accomplished primarily by asynchronous e‐mail communications;  **Contribution of parents/family:** yes  **Contribution of health care practitioners:** yes (face-to-face biweekly counseling sessions) | web-based | yes | **Content:** health education in a coordinated program between face‐to‐face sessions and links to a variety of web sites promoting healthy lifestyle. **Contribution of parents/family:** yes | 24 months | 6 months; 12 months; 18 months; 24 months | child and parent weight; BMI; BMI percentile; body fat | eating behaviors | yes |
| **Williamson et al [21]** | USA | 57 (29/28) | 0/57 | 13.2  (1.4) | **Type:** web-based weight management programs plus face-to-face counselling; **Scope:** weight loss via nutrition education; **Short description:** the web site provided nutrition education, interactive components such as weight graphs and behavior modification for adults and adolescents using a family‐oriented format. Counseling for behavior modification was accomplished primarily by asynchronous e‐mail communications;  **Contribution of parents/family:** yes  **Contribution of health care practitioners:** yes (face-to-face biweekly counseling sessions) | web-based | yes | **Content:** health education in a coordinated program between face‐to‐face sessions and links to a variety of web sites promoting healthy lifestyle. **Contribution of parents/family:** yes | 6 months | 6 months; 12 months; 18 months; 24 months | child and parent weight; BMI; BMI percentile; body fat | na | yes |
| **^a^**Technology-based intervention combined with conventional care.  ^b^SBP: systolic blood pressure.  ^c^DBP: diastolic blood pressure.  ^d^LDL-C: low density lipoprotein cholesterol.  ^e^BMD: bone mineral density.  ^f^HDL-C: high density lipoprotein cholesterol.  ^g^BFC: Big Friends Club.  ^h^SDS: SD score.  ^i^SB2: Student Bodies.  ^j^N/A: not applicable. | | | | | | | | | | | | | |
