# Supplementary material for: Digital Health Interventions for Weight Management in Children and Adolescents: Systematic Review and Meta-analysis
Source: J Med Internet Res. 2022 Feb 14;24(2):e30675. doi: 10.2196/30675 (PMC8887634; doi:10.2196/30675)
Supplement: Multimedia Appendix 2 [file jmir_v24i2e30675_app2.docx]

| **Table 2** Primary and secondary outcomes of the eligible clinical trials of the meta-analysis (n=9; 8 studies) | | | | | | | | | |
| --- | --- | --- | --- | --- | --- | --- | --- | --- | --- |
| **Study** | **Weight-related measurment** | **Weight-related results** | **Significance in BMI metric** **differences between groups** | **Other anthropometric metrics** | **Dietary intake and dietary behaviors** | **Physical activity level and sedentary behaviors** | **Psychological and quality of life assessment** | **Clinical and biochemical metrics** | **Satisfaction for the technology-based intervention** |
| **Chen et al [16]** | mean change in BMI | **Intervention 0:** 27.37 (3.26); **3 months:** 26.91 (3.25); **6 months:** 26.93 (3.43) | no | N/A | ↓ sugary beverages | ↓ screen time | ­↑ self-efficacy in nutrition and physical activity | N/A | N/A |
|  |  | **Control 0:** 28.35 (4.36); **3 months:** 28.81 (4.43); **6 months:** 29.18 (3.88) |  |  |  |  |  |  |  |
| **Vidmar et al [17]** | mean change in BMI z-score | **Intervention 0:** 2.20 (0.31); **3 months:** 2.12 (0.32); **6 months:** 2.10 (0.31) | no | N/A | N/A | N/A | ­↑↓ (lower food addictive behaviors in intervention group compared with the baseline yet non-significant difference, in 6 months) | N/A | More than 500 times use of app over the 6-month period |
|  |  | **Control 0:** 2.39 (0.34); **3 months:** 2.34 (0.36); **6 months:** 1.90 (0.30) |  |  |  |  |  |  |  |
| **Staiano et al [18]** | mean change in BMI z-score | **Intervention 0:** 2.06 (0.46); **24 weeks:** | yes | ↑↓ body fat | ↓ carbohydrates | ­↑↓ moderate to vigorous physical activity | ­ ↑self-efficacy in physical activity | ↓ SBP, DBP, CHOL, LDL-C | 94.4% intervention adherence with high rates of acceptability and enjoyment |
|  |  | **Control 0:** 2.10 (0.42); **24 weeks:** |  |  |  |  |  |  |  |
| **Wright et al [19]** | mean change in BMI | **Intervention 0:** 26 (2.3); **12 weeks:** 26 (2.60) | no (within intervention group: greater reduction in intervention users vs. non-users accompanied by lower energy intake) | N/A | ­↑ fruits consumption | ­↑↓ | ­ ↑ self-efficacy in nutrition; ­↑↓ self-efficacy in physical activity | N/A | moderate |
|  |  | **Control 0:** 25.3 (2.2); **12 weeks:** 25.5 (2.40) |  |  |  |  |  |  |  |
|  | mean change in BMI z-score | **Intervention 0:** 1.90 (0.24); **12 weeks:** 1.90 (0.28) | no (within intervention group: greater reduction in intervention users vs. non-users accompanied by lower energy intake) | no |  |  |  |  |  |
|  |  | **Control 0:** 1.90 (0.29); **12 weeks:** 1.90 (0.30) |  |  |  |  |  |  |  |
| **Nguyen et al [23]** | mean change in BMI | **Intervention 0:** 30.8 (4.20); **12 months:** 31.4 (4.80); **24 months:** 30.8 (4.60) | no | ↓ waist-to-hip ratio (in 24 months) | ↓ high fat meat products; ­ ↑ daily lunch; ↓ fruit juice consumption | ↓ light intensity physical activity; ↑­ total and non-screened leasure time activities | ­↑ body shape satisfaction; ↑­ subjective social status; ↑­ global self-worth | ­↑ SBP (in 24 months) | N/A |
|  |  | **Control 0:** 30.8 (3.50); **12 months:** 30.8 (3.80); **24 months:** 31.8 (4.50) |  |  |  |  |  |  |  |
|  | mean change in BMI z-score | **Intervention 0:** 2.03 (0.37); **12 months:** 1.97 (0.42); **24 months:** 1.83 (0.51) | yes (in 24 months) |  |  |  |  |  |  |
|  |  | **Control 0:** 2.02 (0.29); **12 months:** 1.94 (0.32); **24 months:** 1.93 (0.39) |  |  |  |  |  |  |  |
| **de Niet et al [24]** | mean change in BMI-SDS | **Intervention 0:** 2.63 (0.45); **3 months:** 2.46 (0.54); **6 months:** 2.40 (0.59); **9 months:** 2.36 (0.62); **12 months:** 0.38 (0.59) | yes (in 12 months) | N/A | ­↑ adherence to a healthy eating pattern (the first 3 months) | ­↑ athletic competence | ­↑ global self worth; ↑ psychological health | N/A | children who received short message service were less likely to withdraw from the family-based behavioural lifestyle BFC treatment than children who did not receive the short message service |
|  |  | **Control 0:** 2.54 (0.44); **3 months:** 2.39 (0.52); **6 months:** 2.30 (0.56); **9 months:** 2.42 (0.59); **12 months:** 2.34 (0.57) |  |  |  |  |  |  |  |
| **Doyle et al [20]** | mean change in BMI z-score | **Intervention 0:** 2.19 (0.50); **16 weeks:** 2.11 (0.51) | yes (no retention of significant difference in 4-month follow-up) | N/A | ­↑ dietary restraints (no retention of significant difference in 4-month follow-up) | N/A | ­ ↑ self-efficacy in nutrition and physical activity (retention of significant difference in 4-month follow-up) | N/A | 79% were satisfied with the intervention |
|  |  | **Control 0:** 2.19 (0.44); **16 weeks:** 2.20 (0.43) |  |  |  |  |  |  |  |
| **Williamson et al [22]** | mean change in BMI | **Intervention 0 to 24 months:** + 0.73 (0.66) | no | ↓ body fat; ↓ parent BMI | ↓ high fat foods | ­¯ | ­¯ | N/A | similar drop-out rates in control and intervention group |
|  |  | **Control 0 to 24 months:** +1.20 (0.65) |  |  |  |  |  |  |  |
| **Williamson et al [21]** | mean change in BMI | **Intervention 0:** 35.31 (7.6); **6 months:** | yes | ↓ body fat; ↓ parent BMI | ↓ high fat foods | ­¯ | ↓ dieting and weight concerns | N/A | similar drop-out rates in control and intervention group |
|  |  | **Control 0:** 37.34 (8.16); **6 months:** |  |  |  |  |  |  |  |

^a^N/A: not applicable.

^b^SBP: systolic blood pressure.

^c^DBP: diastolic blood pressure.

^d^CHOL: total cholesterol.

^e^LDL-C: low density lipoprotein cholesterol.

^f^SDS: SD score.

^g^BFC: Big Friends Club.
